# Hydroethanolic Extract of *Solanum paniculatum* L. Fruits Modulates ROS and Cytokine in Human Cell Lines

**DOI:** 10.1155/2020/7240216

**Published:** 2020-01-22

**Authors:** Ana Paula C. R. Ferraz, Alessandra Sussulini, Jéssica L. Garcia, Mariane R. Costa, Fabiane V. Francisqueti-Ferron, Artur J. T. Ferron, Carol Cristina V. de A. Silva, José Eduardo Corrente, Vanessa M. Manfio, Vickeline Namba, Giuseppina P. P. Lima, Bismarque S. Pereira, Denise Fecchio, Igor O. Minatel, Klinsmann C. dos Santos, Camila R. Corrêa

**Affiliations:** ^1^São Paulo State University (UNESP), Medical School, Botucatu 18618-687, Brazil; ^2^University of Campinas (UNICAMP), Institute of Chemistry, Campinas 6154, Brazil; ^3^São Paulo State University (UNESP), Institute of Biosciences, Botucatu, 18618-689 São Paulo, Brazil

## Abstract

*Solanum paniculatum* L. or popularly known as “jurubeba” is an herbal medicinal plant. A few studies have investigated its biological effects; however, research aimed at elucidating the redox balance effects from its fruits has not been reported so far. ROS interplays in various fields of medicine such as chemotherapy. Here, we evaluated antioxidant and inflammatory activities of the hydroethanolic extract of *Solanum Paniculatum* L. (HESPL) fruits in breast cancer cells, as well as its phytochemical profile. The antioxidant profile (carotenoids and phenolic compounds) was obtained by HPLC-DAD-UV and HPLC-APCI-MS. Cancer cell lines and human vein endothelial cells (HUVECs) were cultivated and treated with 1.87-30 *μ*g/mL of HESPL for 24 hrs. Cytotoxicity, oxidative, and inflammation biomarkers were evaluated. The dose of 30 *μ*g/mL of the HESPL extract presented cytotoxicity in the MCF-7 cell line. However, for MDA-MB-231, the cytotoxicity was observed in the dose of 1.87 g/mL. The 1.87 *μ*g/mL and 3.75 *μ*g/mL doses decreased the concentration of IL-6 in MCF-7 cells. In the MDA-MB-231 cells, the HESPL did not decrease the IL-6 concentration; however, in the doses of 15 and 30 *μ*g/mL, an increase in this parameter was observed. The HESPL increased IL-1*β* concentration in HUVECs. The ROS level in MCF-7 was elevated only at the 30 *μ*g/ml dose. Regarding MDA-MB-231, HESPL promoted increased ROS levels at all doses tested. HUVEC showed no increase in ROS under any dose. HESPL treatment may modulate cytotoxicity, ROS, and cytokine levels due to its phytochemical profile, and it has shown an antioxidant or anti-inflammatory effect.

## 1. Introduction

Cancer is a multidimensional and complex onset of diseases with deregulated mechanisms and biochemical signaling, leading to pathology progress in a biological system [1]. Among all cancers, breast cancer is the main cause of worldwide women's death; therefore, it is a target for research studies involving early diagnosis detection and therapies [2].

ROS, a type of unstable molecules that contain oxygen, which are rapidly transformed into other species can induce oxidation of free amino acids, residues, and proteins. On the other hand, they can be a target of multiresistant drugs, enhancing cell death [3]. These species play a role in various fields of biology and medicine of cancer on protumorigenic signaling, cell proliferation, and tumorigenesis and transcription factor activation, which in turn can promote cytokines and chemokines such as IL-6 production in inflammation pathways as well as regulating and inducing apoptosis [4]. These reactive species were counteracted or stimulated by substances known as antioxidants, acting on the endogenous cellular and exogenous environments, or by interactions of low molecular mass antioxidants such as carotenoids and phenolic compounds [5].

Natural products from medicinal and nonconventional plants and functional food intake have also been reported by data, which prevent the onset of several diseases or are used as a treatment for several diseases, providing ethnopharmacological knowledge on nutrition therapeutic formulations containing antioxidants on their phytochemical profile [6]. Several studies show their effectiveness in modulating ROS, inflammation, and chemotherapeutic resistance [7, 8].

*Solanum paniculatum* L. (*Roem. and Schult.*), *Solanaceae* family, popularly known as “jurubeba,” “jurupeba,” “jubeba,” or “juna” is an unconventional fruit-vegetable native to Tropical America. Its leaves and roots are widely used in traditional Brazilian medicine as tonic eupeptic agents to treat gastric and liver dysfunctions [9]. Its fruits are consumed by decoctions in culinary preparations and with oil or vinegar on pickled jurubeba [10]. Evidence has reported the presence of many steroidal compounds such as glycoalkaloids and saponins [11] and *β*-sitosterol [12] used in folk medicine. Endringer and colleagues showed chemoprevention in liver cells via NF-*κ*B inhibitory activity after *Solanum paniculatum* L. treatment [13]. On the other hand, the study of Rios et al. (2017) showed its potential treatment of inflammatory conditions, reducing cell proliferation, IL-4, NO production, and other inflammatory markers; however, no chemical investigation of the antioxidant profile of *Solanum paniculatum* L. fruits as an hydroethanolic formulation and its biological mechanisms in breast cancer has been reported so far. These evaluations provided by HESPL response could be important on ROS and cytokine pathway knowledge. The present study was undertaken to investigate the phytochemical profile of HESPL fruit formulation and its effects on ROS and cytokine production in human breast cancer and endothelial cells.

## 2. Materials and Methods

### 2.1. Plant Material

*Solanum paniculatum* L. (*in natura*) fruits were collected on December 31, 2017, in the south of Mato Grosso State (Campo Grande region), with the coordinates 20°47′27^″^S latitude and 54°56′86^″^S longitude, by Ana Paula Costa Rodrigues Ferraz; these were deposited to the herbarium at the Institute of Biosciences, São Paulo State University (UNESP), Botucatu, SP, Brazil, under the voucher number 33072. Fruiting ratios for jurubeba were established according to Nurit et al. [14] and Forni-Martins et al. [15] through its globulous greenish to yellow appearance releasing from a peduncle; after this, 392 g of fruits was separated according to their appearance, washed in water, and stored at -80°C until the extraction process.

### 2.2. Preparation of the HESPL Extract

Jurubeba fruits were macerated in a cryogenic mill (6775 Freezer/Mill® Cryogenic Grinder), and lyophilized in a lyophilizer (Liotop L108®), obtaining a dry weight of 129 g of the powdered extract. The powdered extract (109 g) was percolated by exhaustion according to the Brazilian Homeopathic Pharmacopoeia (2011) with a slow rate of 3 mL/min/kg using 70% ethanol. After twenty-four days of percolation, solvents were evaporated in a vacuum rotating evaporator with a horizontal condenser (Marconi MA-122) under low pressure (45°) and the remaining liquid was lyophilized to obtain the HESPL crude extract (24.12 g). The yield of the extract was calculated by

(1) $$\begin{array}{c} x=\left(\frac{\text{final weight}\left(\text{g}\right)}{\text{dry weight }\left(\text{g}\right)}\right)\times 100. \end{array}$$

### 2.3. Extraction of Carotenoids and Vitamin E via HPLC-DAD-UV and HPLC-APCI-MS

The hydroethanolic extract (100 mg) was subjected to basic hydrolysis to separate the liposoluble components using 30% KOH in ethanol and solubilized with ether/hexane (2 : 1), using the saponification method adapted for plant samples from Qin et al. (1997). For HPLC-DAD-UV analysis, aliquots of 20 *μ*L were injected into the Waters Alliance 2695e Separation Module (Waters Corporation, Milford, MA, USA) coupled with the Waters PDA 2998 detector and analytical C_30_ column (Thermo Scientific—3 *μ*m (4.6 × 150 mm)). The mobile phases were composed of methanol/methyl tert-butyl ether/water: (A) 85/12/3 (*v*/*v*/*v*) and (B) 8/90/2 (*v*/*v*/*v*), with 6 mmol/L of ammonium acetate. The gradient was 2 min at 5% B, 3 min at 10% B, 6 min at 15% B, 10 min at 25% B, 12 min at 40% B, 16 min at 83%B, 20 min at 95% B, 24 min at 95% B, 26 min at 40% B, 30 min at 5% B, and 32 min at 5% B with a flow rate of 1.0 mL/min. Complementary to these data and with the same method for sample extraction (2.5 mg), HPLC-APCI-MS analysis of carotenoids was determined. Aliquots of 10 *μ*L were injected into an Agilent 1290 Infinity High-Performance Liquid Chromatography system (Agilent Technologies, United States) adapted by Etzbach et al. for total carotenoids, performed on 40-minute run and at 0.5 mL/min flow rate. The eluent B proportion was modified for 30/60/10 (*v*/*v*/*v*) using the same HPLC-DAD-UV column previously described [16].

#### 2.3.1. HPLC-APCI-Mass Spectrometry (MS) Instrumentation and Carotenoid Identification

Mass spectrometry was performed in an AB Sciex Triple Quad™ QTRAP® 5500 Mass Spectrometer equipped with an Atmospheric-Pressure Chemical Ionization (APCI) source on a positive mode for carotenoid analysis. Also, 5 mmol/L of ammonium acetate was added to the mobile phases for improving compound ionization, as well as the column temperature (25°C). The conditions for mass spectrometry were adapted from Etzbach et al. (2018) with the following modifications: entrance potential (EP), 10 V; collision energy (CE), 30 V; collision cell exit potential (CXP), 8 V; time, 50 ms; curtain gas, 10 (API); medium collision gas (CAD); ion spray voltage, 5500 V; temperature, 450°C; and arbitrary units for ion source gas 1 (GS1), 30.0.

A selective reaction monitoring (SRM) experiment was performed to identify the analytes, the first mass transition following two or three mass transitions was used to confirm the compound profile, and these transitions were determined by the literature [16] (see Table 1).

The major carotenoids (see Figure S1 in Supplementary Materials) constituent from HPLC-DAD-UV were focused on these evaluations, and the SRM identification transition selected was 569.00/551.00. The Analyst® 1.5.1 software (AB Sciex®) and MultiQuant™ 3.0.3 software (AB Sciex®) were used for data analysis.

See Figure S1 in Supplementary Materials for comprehensive carotenoid image analysis.

### 2.4. Extraction and Identification of Phenolic Compounds via HPLC-DAD-UV

The HESPL extraction (100 mg) was determined by modifications of the method described by Palafox-Carlos et al. [17]. The extract was dried in N_2_, resuspended in HPLC-grade methanol, and filtered on a 0.22 *μ*m Analytical® nylon membrane. Aliquots (20 *μ*L) were injected into a UHPLC Thermo Scientific Dionex UltiMate 3000 system (Thermo Fisher Scientific Inc., MA, USA), coupled with a quaternary pump, an Ultimate 3000RS autosampler, and a diode array detector (DAD-3000RS) using a C_18_ column, and the flow rate was 0.8 mL/min. The mobile phase was composed of phosphoric acid/water (A) (0.85/99.15, *v*/*v*) and acetonitrile (C) (100, *v*). The gradient was 2 min at 7% B, 3 min at 9% B, 5 min at 10% B, 7 min at 12% B, 8.5 min at 13% B, 11 min at 15% B, 12.5 min at 20% B, 13 min at 21% B, 14 min at 23% B, 15 min at 25% B, 17 min at 30% B, 19 min at 45% B, 22 min at 65% B, 23 min at 75% B, and 24 min at 30% B. See Figure S2 in Supplementary Materials for comprehensive phenolic compound image analysis.

### 2.5. Antioxidant Capacity

#### 2.5.1. Radical Sequestration Method (DPPH^·^) and FRAP Assay

A methanolic solution was prepared for the extraction with 0.100 g of jurubeba for both methods. The DPPH method was performed according to Brand-Williams et al. [18], and the FRAP assay was adapted according to the methodology proposed by Benzie and Strain [19], which is efficient in the determination of antioxidant activity by iron reduction.

### 2.6. Cell Culture Experimental Design

Breast cancer cell lines were MCF-7 (ATCC® HTB22™), luminal, HER2+, and estrogen- and progesterone-positive receptors and MDA-MB-231 (ATCC® HTB26™), basal and with invasive potential. MCF-7 were cultivated in RPMI Medium 1640 supplemented with 10% FBS, 1% NEAA, 1% sodium pyruvate, and 1% antibiotic and MDA-MB-231 were cultivated with 10% FBS and 1% antibiotic (anti-anti). HUVECs (ATCC® CRL-1730) were cultivated in F-12K Medium (Thermo Fisher Scientific® Gibco DMEM/F12 DULBEC) with 10% FBS and 1% antibiotic. All cell lines were maintained in a humidified incubator (5% CO_2_ at 37°C). Cells were plated onto 175 cm^2^ tissue culture flasks, and treatments proceeded when the cells reached 80% of confluence between passages 6 and 8. A stock solution for HESPL induction (10 mg/mL) was made in DMSO as a vehicle according to recommendations by Jamalzadeh et al. (2016) for an experimental application. Different doses (30, 15, 7.5, 3.75, and 1.87 *μ*g/mL) were established by Rios et al. (2017) and were diluted in medium without FBS for further *in vitro* experimental research.

The cell viability was performed by the Trypan Blue assay. After 24 hours of treatment, cells were harvested and submitted to trypsinization, the pellet was resuspended in appropriate concentration of medium, and 10 *μ*l was collected and diluited in 10 *μ*l of Trypan Blue. The cells were counted on an improved Neubauer Haemocytometer (Weber Scientific International Ltd., UK). Viable and nonviable cells were counted under light microscopy, and the viable cells are considered above 80%.

### 2.7. Cytotoxic Activity

Approximately 1 × 10^6^ cells were incubated on six-well plates for 12 hours on RPMI 1640 culture medium with 10% FBS and 24 hrs with 0.1% FBS and maintained at 37°C and an atmosphere of 5% CO_2_. Different doses were applied for 24 hrs aimed at providing a nonlethal dose for further research. Subsequently, cytotoxicity was determined using the rapid colorimetric assay based on the tetrazolium salt MTT (3-(4,5-dimethylthiazol-2-yl)-2,5-diphenyl tetrazolium bromide) (0.5 mg/mL in HBSS) which can measure metabolic living cells and proliferation [20]. The cells were solubilized in DMSO and read at 570 nm using a scanning multiwell spectrophotometer (SpectraMax 190, Molecular Devices).

### 2.8. Analysis of ROS Production

The cells were cultivated onto 75 cm^2^ flasks for each dose, and following the treatments, these were collected using trypsin and resuspended on Muse™ Oxidative Stress Assay Buffer (4700-1330, Merck Millipore) with a minimum of 1 × 10^6^ cells for each replicate; these were performed through flow cytometry using Muse® Cell Analyzer (Merck, Darmstadt, Germany) with Muse™ Oxidative Stress Kit (MCH100111, Merck Millipore). The results were shown positively ROS (ROS (+)) which has the cell exhibiting ROS significance.

### 2.9. Inflammation Measurement

IL-6 and IL-1*β* evaluations were performed in a commercial immunoassay ELISA kit using 100 *μ*L of cell supernatant according to the manufacturers' instructions (Linco Research Inc., R&D Systems®, Millipore, and B-Bridge International Inc.) and were determined by absorbance using a microplate reader (SpectraMax 190, Molecular Devices).

## 3. Statistics

Results are expressed as mean ± SD. One-way ANOVA followed by the Tukey test was performed by normal distribution in SigmaPlot using Windows 10. Statistical significance was considered when *p* < 0.05.

## 4. Results

### 4.1. HESPL Extract and Bioactive Compounds

The results for a phytochemical profile of HESPL in HPLC-DAD-UV presented with four carotenoids (lutein, zeaxanthin, *β*-cryptoxanthin, *β*-carotene), one vitamin E (*γ*-tocopherol), two phenolic compounds (chlorogenic and caffeic acids), and one flavonoid (quercetin) (see Table 2).

**Figure 1:**
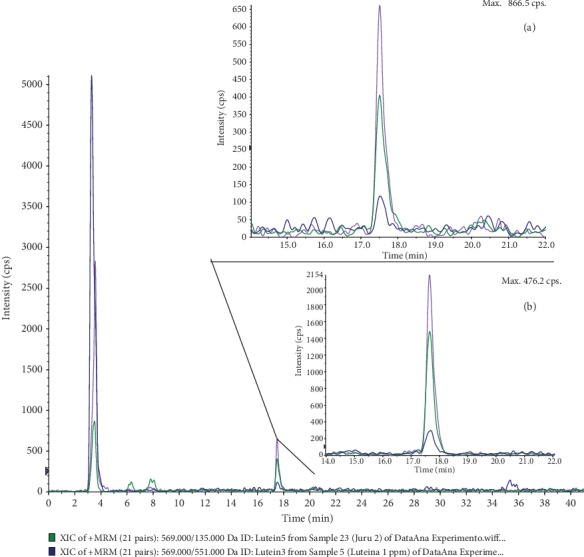
Carotenoid profile of *Solanum paniculatum* L. by HPLC-APCI-MS: (a) lutein compound in the sample; (b) lutein standard.

The major active compound in HESPL is lutein, identified for the retention time of 17.52 min based on similar [M+H]+ (*m*/*z*) (569.0) and fragments (175/135/551) on a sample (Figure 1(a)) compared with the lutein standard (Figure 1(b)).

HESPL has a yield of 22.19%. The FRAP procedure measures the antioxidant capacity through the interaction between the reductants (antioxidants) and Fe^II^-TPTZ creating a blue color showing 423.32 ± 1.70 *μ*mol of quercetin 100 g^−1^ DW (CV = 0.40). Additionally, the DPPH method has an affinity of reducing a free radical by a hydrophilic affinity, and HESPL shows 89.13 ± 1.20 (mg of quercetin 100 g^−1^) (CV = 1.34) of % DPPH reduction.

### 4.2. Cell Viability of the Cell Lines Submitted to Different Doses

**Figure 2:**
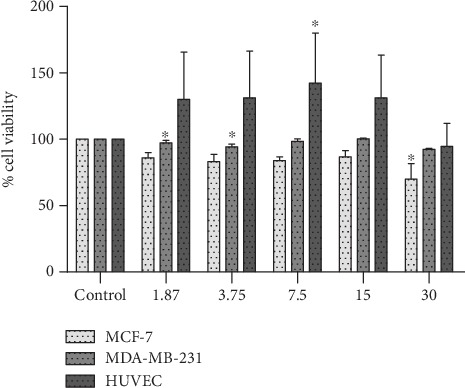
% cell viability from MCF-7, MDA-MB-231, and HUVECs. ^∗^Versus control when *p* < 0.05. The viability was determined by the MTT assay. The dose of 30 *μ*g/mL presented cytotoxicity.

The dose of 30 *μ*g/mL (82.29 ± 19.11, *CV* = 23.22) shows a significant toxicity (*p* < 0.05) when compared with control in MCF-7 cells (see Figure 2). However, for MDA-MB-231, the cytotoxicity was observed in the dose of 1.87 *μ*g/mL (97.22 ± 2.04, *CV* = 2.10). Regarding the HUVEC, the extract did not show cytotoxicity.

### 4.3. Concentration of Interleukin in the Cell Lines in Different Doses of HESPL

**Figure 3:**
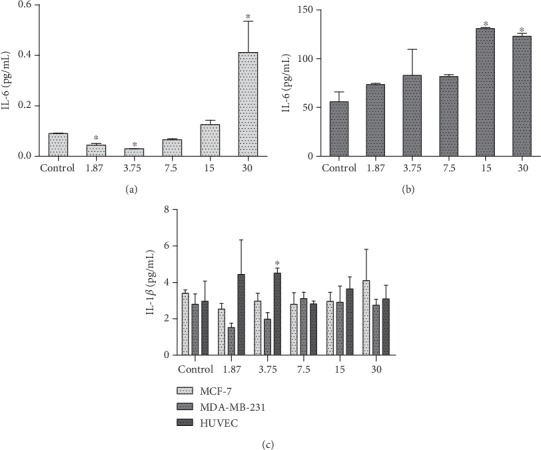
Different doses of HESPL effect in interleukin-6: (a) MCF-7 cells, (b) MDA-MB-231 cells, and (c) different doses of HESPL effect in IL-1*β* in the cell lines. ^∗^Versus control when *p* < 0.05. The dose of 3.75 *μ*g/mL diminished significantly compared with other doses. On the other hand, the doses of 15 and 30 *μ*g/mL were increased in MDA-MB-231, and for IL-1*β*, the dose 3.75 *μ*g/mL was enhanced.

In this study, the 1.87 *μ*g/mL (0.04 ± 0.02, *p* < 0.005) and 3.75 *μ*g/mL (0.03 ± 0.00, *p* < 0.005) concentrations decreased with significance in MCF-7 cells (Figure 3(a)). In the MDA-MB-231 cells, the HESPL did not decrease the IL-6 concentration; however, in the doses of 15 and 30 *μ*g/mL, an increase in this parameter was observed (130.88 ± 1.52/125.46 ± 4.88, *p* < 0.005) (Figure 3(b)). The HESPL increased IL-1*β* concentration in HUVECs (dose of 3.75 *μ*g/mL (4.513 ± 0.280, *p* < 0.05) vs. control (2.965 ± 1.108); Figure 3(c)).

### 4.4. The Levels of ROS Were Increased by the Dose of 7.5 *μ*g/mL of HESPL vs. Control

**Figure 4:**
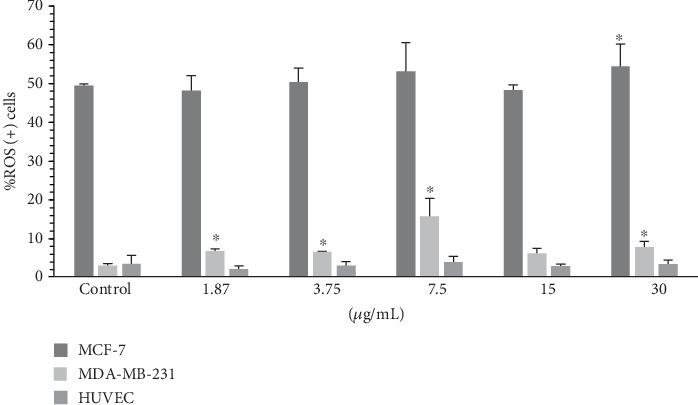
ROS parameter from MCF-7, MDA-MB-231, and HUVECs. ^∗^Versus control when *p* < 0.05. The ROS was enhanced in all doses tested in invasive cells, and the dose 30 *μ*g/mL was increased in MCF-7 cells.

**Figure 5:**
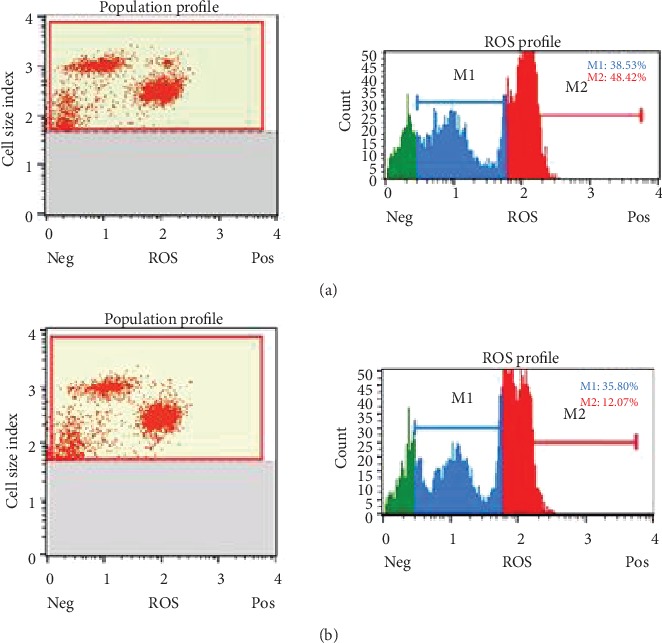
Cell flux analysis in MCF-7 cells: (a) 30 *μ*g/mL treatment; (b) control.

The higher dose of 7.5 *μ*g/mL of HESPL improves ROS levels (see Figure 4(a)) in MDA-MB-231 (15.47 ± 4.88, *p* < .0001) vs. control (2.87 ± 0.57) and also improved increased levels in all tested doses. HUVECs did not present higher levels of ROS. See Figure 5 for a comprehensive example of cell flux analysis.

## 5. Discussion

Dietary phytochemicals can act as an antioxidant or prooxidant and may participate in the development of new anticancer drugs [21]. Elevated levels of ROS and deregulated redox signaling are common hallmarks of cancer progression and resistance to treatment [22]. ROS production is involved in two faces of the cancer environment: in basal levels, these species are involved in PI3K/Akt-mediated cell survival and proliferation, or when excessive intracellular ROS accumulation occurs, these are involved in the cleavage of caspase-3 and caspase-7 also damaging nucleic acid bases and other compounds [4].

It is known that MDA-MB-231 are malignant cells, and in this study, they could be a great model to investigate the effects of natural products on ROS modulation since this kind of cell is classified with invasive potential [23]. Our dose-response study demonstrated that the dose ranging from 1.87 to 7.5 *μ*g/mL of HESPL enhanced ROS production (Figure 4). These results suggest that the phytochemical profile of plant- and food-based diets may act on the redox balance. Phytochemicals present in plants can act as a prooxidant via the Fenton reaction whose mechanism occurs via iron-dependent ROS production [24]. It can also inhibit NADPH expression and the blockade of the Nrf-2 signaling pathway [25]. This transcription factor was commonly elevated in various types of cancer and consequently as a result of the activation of oncogenes such as K-Ras, B-Raf, and c-Myc which in turn are involved in survival and cell proliferation [21].

The mechanism of action of plant-based diets may possess intervention capacity acting at specific pathways such as reduction of NF-*κ*B DNA-binding activity, TNF-*α* inhibition, increased caspase-3 and caspase-7, bax/bcl-2 ratio, and fraction with sub-G0/G1 DNA content in apoptosis [26]. Additionally, Sinha et al. demonstrated that phytoconstituents of tea (*Camellia sinensis*) modulate epidermal growth factor receptor, B-cell lymphoma 2 (Bcl-2), and Bcl-2-associated X protein in the breast carcinoma [27].

The phytochemical profile of the jurubeba fruit extract consists of phenolic compounds, vitamin E, and carotenoids. Polyphenols can act via noncovalent interaction with cellular proteins promoting the inhibition of prooxidant enzymes and diminishing DNA damage and lipid peroxidation as well as inhibition of ROS-dependent signal transduction [28]. In our phytochemical profile, caffeic acid is the major phenolic compound, but we also identified chlorogenic acid and quercetin. Yu et al. demonstrated that caffeic acid can lead to apoptosis in YD-15 (human mucoepidermoid carcinoma), HSC-4, and HN22 (human oral squamous cell carcinoma). Moreover, the authors have shown the apoptotic effect by cleavages of caspase-3 and poly (ADP-ribose) polymerase and activation of Bax protein [29]. In parallel, Abou-Hashem et al. showed the apoptotic effect of chlorogenic acid via cell cycle arrest at the sub-G0 phase and DNA fragmentation [30], whereas quercetin can induce apoptosis through proteasome inhibition such as the 20S and 26S proteasome in Jurkat T cells and accumulation of polyubiquitinated proteins [28].

Evidence suggests that dietary carotenoids may help in reducing the risk of breast cancer [31]. Lutein is the major carotenoid identified in our extract. Lutein acts by suppressing inflammation, and it was involved in the inhibition of NF-*κ*B signaling [32]. In summary, benefits of lutein intake consist in eye health and antioxidant and anti-inflammatory activities. It is suggested that lutein mechanisms of action in cancer might be involved in cell growth inhibition by inducing cell cycle arrest and caspase-independent cell death also, activating p53 signaling [33]. Juin et al. showed the apoptotic effect of zeaxanthin through the expression of the BRAF V600E oncogene [34]. Additionally, Gao et al. demonstrated the antiproliferation effect of *β*-cryptoxanthin by G0/G1 cell cycle arrest and AMPK signal inactivation [35]. The effects of *β*-carotene on metastasis was evidenced by Kim et al. where it downregulated the expression of CSC markers, MMPs, and HIF-1*α* in cancer tissues [36]. Inhibition of the HMG-CoA reductase enzyme and inhibition of the NF-*κ*B pathway are the main antiangiogenic mechanisms found for tocotrienols such as *γ*-tocopherol [37].

Cytokine levels are naturally enhanced in conditions such obesity and cancer [38]. A few reports related the reduction of IL-6 to a targeted therapy for cancer [39, 40]. In our study, the HESPL treatment diminished the levels of IL-6 in MCF-7 cells (Figures 3(a) and 3(b)), enhancing the importance of this cytokine acting as having multifaceted cellular displayed and physiological functions on biological systems [41]. Primary tumors such as MCF-7 cells may be an important tool on research focusing on chemopreventive actions [42]. The chemopreventive actions can attenuate cell cycle arrest [43] and other various tumor-promoting pathways in cancer [44]. IL-6 seems to be an important cytokine in breast cancer studies [39, 44, 45] acting in several pathways involved in cancer progression [46]. Moreover, IL-1*β*, another important cytokine in the chronic inflammation process, was enhanced in HUVECs after treatment with 3.75 *μ*g/mL HESPL (Figure 3(c)).

The synergic effects of different compounds presented in the HESPL might provide an adjuvant strategy on cancer therapies since multiple phytochemicals may act on various biological microenvironments. We have shown in this study that phytochemicals presented in the crude extract of jurubeba fruits can act on the oxidative and inflammation balance. A positive control study may be a scientific contribution for our current findings since the isolated phytochemicals contribute to antitumoral mechanisms such as apoptosis. However, its known that individual compounds have not shown benefits in some clinical trials since bioactivity might be affected or do not react in the same effective way when compared to crude extracts of plant-based foods [26]. Our data suggests a redox/inflammation modulation network of the *Solanum paniculatum* L. fruits, and its potential as a nutraceutical was shown to be enhanced as it successfully modulates ROS and cytokine production as well as inhibiting the growth of cancerous cells.

## 6. Conclusions

The molecular mechanisms of ROS and cytokine play an important role in various cancer models evidenced in both *in vitro* and *in vivo* studies. ROS and cytokine modulation seems to be a promising chemotherapeutic target for treatments. Here, we demonstrated that HEPSL can act by diminishing ROS production and modulate levels of IL-6 and IL-1*β*. Most importantly, our dose-response studies demonstrated that different concentrations of phytochemicals, acting synergically, might affect the outcomes either positively or negatively, raising the importance of ingestion-response ratio effects of diets. Our findings, even as a basic research model, provide information that can guide future studies aimed at elucidating new therapeutic alternatives for cancer.

## Figures and Tables

**Table 1:** SRM transitions for total carotenoids.

Compound name	[M+H]+ (*m*/*z*)	Fragment ion	Declustering potential (V)
Lutein	569.0	476.0	80
		175.0	
		551.0	
		533.0	

Zeaxanthin	569.0	551.1	50
		533.1	
		395.0	
		93.0	
		135.1	

*β*-Cryptoxanthin	553.5	119.0	50

*β*-Cryptoxanthin	553.0	135.0	50
		495.0	
		461.0	

*α*-Carotene		123.0	
	537.0	481.0	50
		444.1	
		177.2	
		445.4	

*β*-Carotene	537.0	413.3	50
		269.2	

**Table 2:** Phytochemical profile of HESPL determined by HPLC-DAD-UV.

Analyte	RT	Linearity range (ng/*μ*L)	LOD/LOQ (ng/*μ*L)	*r* ^2^	*Solanum paniculatum* L. (pg/mL) in 100 mg of extract	SD (%)	CV (%)
Carotenoids							
Lutein	3.449	5-100	1.45/5	0.9988	103.70	2.3049	2.222719
Zeaxanthin	4.133	2.28-36.5	0.80/2.28	0.9972	8.9	0.48608	5.461573
*β*-Cryptoxanthin	9.017	5.30-84.9	0.97/5.30	0.9963	8.8	0.5913	6.719318
*β*-Carotene	15.666	15.6-250	0.89/15.6	0.9821	8.7	1.1533	0.131655
Vitamin E							
*γ*-Tocopherol	5.333	0.075-2.42	1.09/0.075	0.9999	1.6	0.00206	0.12875
Phenols							
Chlorogenic acid	3.517	10-500	1.40/10	0.9956	17.5	0.7415	4.237143
Flavonoids							
Caffeic acid	6.617	10-500	1.30/10	0.9991	23.9	0.9641	4.033891
Quercetin	19.12	10-500	1.33/10	0.9999	2.9	0.1306	4.503448

## Data Availability

The data used to support the findings of this study are available from the corresponding author upon request.

## Supplementary Materials

Supplementary Materials Figure S1: molecular structures of carotenoids. Figure S2: molecular structures of phenolic compounds.
