# Supplementary material for: Hydroethanolic Extract of Solanum paniculatum L. Fruits Modulates ROS and Cytokine in Human Cell Lines
Source: Oxid Med Cell Longev. 2020 Jan 22;2020:7240216. doi: 10.1155/2020/7240216 (PMC7204104; doi:10.1155/2020/7240216)
Supplement: Supplementary Materials — Figure S1: molecular structures of carotenoids. Figure S2: molecular structures of phenolic compounds. [file 7240216.f1.pdf]

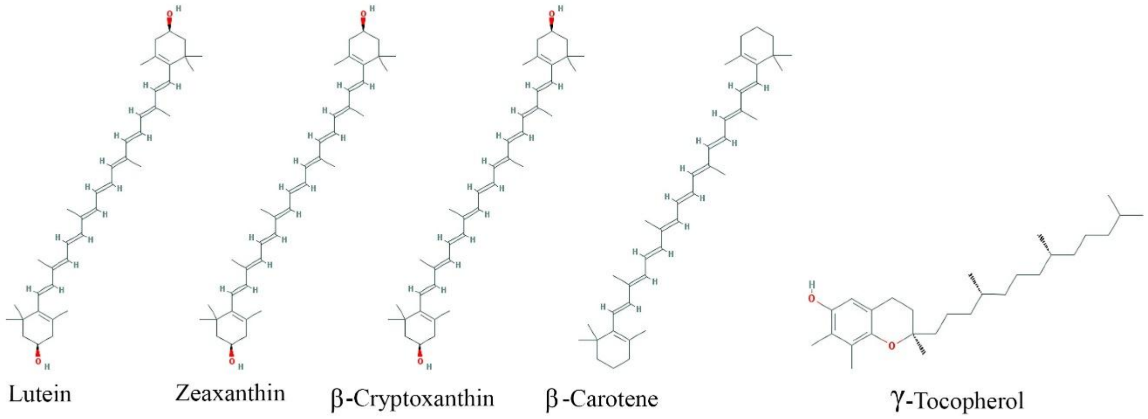

Figure S1: Molecules Structures of Carotenoides.

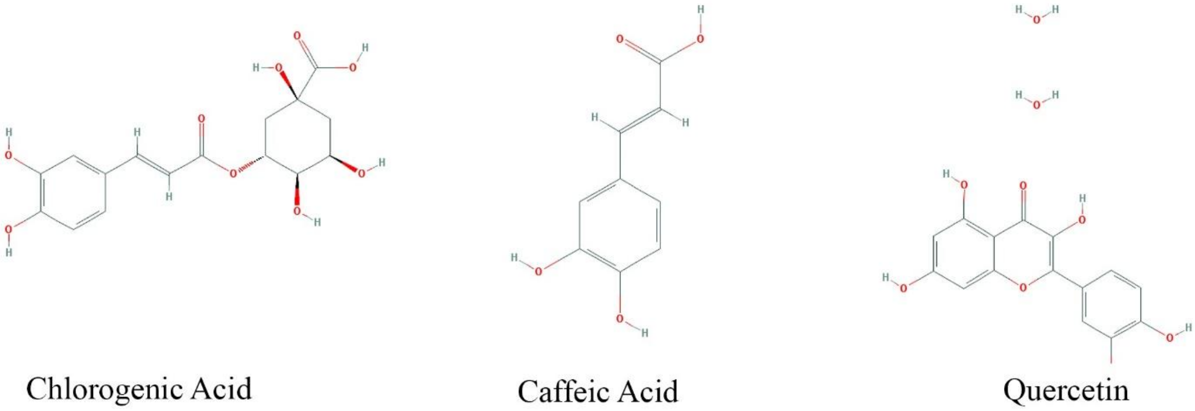

Figure S2: Molecules Structures of Phenolic Compounds.
